# Intra-amniotic sildenafil treatment improves lung blood flow and pulmonary hypertension in congenital diaphragmatic hernia rats

**DOI:** 10.3389/fbioe.2023.1195623

**Published:** 2023-07-20

**Authors:** Shiho Yoshida, Alexander M. Kreger, George K. Gittes

**Affiliations:** Division of Pediatric Surgery, Department of Surgery, UPMC Children’s Hospital of Pittsburgh, University of Pittsburgh School of Medicine, Pittsburgh, PA, United States

**Keywords:** sildenafil, pulmonary hypertension, nitrofen model, congenital diaphragmatic hernia, amniocentesis

## Abstract

Pulmonary hypertension associated with congenital diaphragmatic hernia (CDH) is a critical factor in determining prognosis. We propose that intra-amniotic sildenafil administration is an effective prenatal therapy for CDH-induced pulmonary hypertension. To assess the efficacy of this treatment, we administered sildenafil to nitrofen-induced congenital diaphragmatic hernia fetuses and control fetuses via an intra-amniotic injection after a laparotomy on the pregnant dam at either E13.5 or E15.5. Intra-amniotic sildenafil treatment attenuated peripheral vascular muscularization, enhanced pulmonary blood flow, and increased the ratio of pulmonary artery size to aortic size in congenital diaphragmatic hernia fetuses after both E13.5 and E15.5 treatments. E13.5-treated congenital diaphragmatic hernia fetuses showed a higher and more prolonged expression of cyclic guanosine monophosphate (cGMP)-dependent protein kinase and more production of vascular endothelial growth factor, resulting in a significant improvement in lung architecture. The E13.5-treated congenital diaphragmatic hernia fetuses also had an increase in lung weight-to-body weight ratio and an improved fetal survival. Intra-amniotic sildenafil treatment did not show any detectable negative effects in control fetuses. Intra-amniotic sildenafil treatment for rats attenuates CDH-induced pulmonary hypertension and enhanced peripheral pulmonary blood flow. Moreover, early intervention may be preferable to better accelerate lung development and improve prognosis. Direct sildenafil administration via an intra-amniotic injection may be a promising option in congenital diaphragmatic hernia prenatal therapy.

## Introduction

Congenital diaphragmatic hernia (CDH) is a disease that affects 1 in 2500 live births. It is associated with lung hypoplasia and pulmonary vascular abnormalities, which result in pulmonary hypertension, respiratory distress, and significant morbidity and mortality in the newborn (Leeuwen and Fitzgerald, 2014; Gupta and Harting, 2020). Despite numerous advances in neonatal intensive care unit care, the reported mortality from CDH varies from 30% to 50% based on varying institutional data, plus a significant “hidden mortality” of the unborn fetuses (Cordier et al., 2020; Lakshminrusimha and Vali, 2020). Neonates with CDH may be managed with multiple modalities including extracorporeal membrane oxygenation, inhaled nitric oxide (iNO), and other vasodilators. However, there have been no significant improvements in survival or outcomes with these treatments (Horn-Oudshoorn et al., 2020; Yu et al., 2020). The etiology of CDH has been linked to multiple genetic and environmental factors but remains poorly understood (Leeuwen and Fitzgerald, 2014). Prior studies have suggested that some components of the pulmonary vascular defects are fixed by the time of birth and are not responsive to postnatal pulmonary vasodilator therapy (Gupta and Harting, 2020). Thus, prenatal therapy for CDH has been an area of ongoing investigation (Russo et al., 2019a; Tsao and Johnson, 2020).

Sildenafil, a phosphodiesterase type 5 (PDE5) inhibitor, has been approved by the Food and Drug Administration for use in pulmonary hypertension in adults and is often used as a second-line therapy in persistent pulmonary hypertension (PPHT) in the newborn (Baquero et al., 2006). The drug has also been considered for prenatal treatment of CDH. Several studies have demonstrated improvements in the pulmonary vasculature and lung growth in animal models of CDH following maternal administration of sildenafil (Luong et al., 2011; Yamamoto et al., 2014; Mous et al., 2016; Russo et al., 2016; Kashyap et al., 2019), and clinical trials (SToP-PH) have recently been approved to evaluate the transplacental effects of sildenafil on pulmonary hypertension in CDH fetuses (Russo et al., 2018a). However, this trial had to be halted given the results of the Dutch STRIDER trial that showed an increased incidence of PPHT and neonatal mortality (Groom et al., 2018; Cochius-den Otter et al., 2022). It is questionable whether the STRIDER trial provided sufficient evidence to suspend SToP-PH because it investigated the effect of sildenafil on fetal growth restriction unrelated to CDH (Russo et al., 2019b). Nevertheless, we should consider a different route of administration of sildenafil as prenatal therapy for CDH.

We have proposed direct sildenafil administration to CDH fetuses via an intra-amniotic (IA) injection. Our prior study using a single intra-amniotic injection of sildenafil in the nitrofen-induced CDH mouse model demonstrated an enhancement of embryonic pulmonary blood flow, improvements in the morphology of pulmonary vasculature, and astonishing survival improvements (Okolo et al., 2020). Since intra-amniotic sildenafil is absorbed by the fetus directly, without first crossing the maternal placenta, we believe that intra-amniotic sildenafil treatment may show a much better therapeutic potential for the fetuses and less burden for the mothers than maternal treatment as a prenatal therapy for CDH.

In this study, we aimed to show that intra-amniotic sildenafil would improve not only the abnormal pulmonary vasculature but also lung hypoplasia in an experimental CDH model, with an eye to translation into the clinical setting.

## Material and methods

We evaluated the efficacy of intra-amniotic sildenafil treatment in a rat model of CDH, with a specific assessment of the effects of timing and dosing on the pulmonary phenotype.

All experiments were approved by the Animal Research and Care Committee at the Children’s Hospital of Pittsburgh and the University of Pittsburgh Institutional Animal Care and Use Committee (IS00017834).

### Animal model

CDH fetuses were induced by nitrofen administration to pregnant rats. Pregnant Sprague-Dawley rats were gavage fed 100 mg of the herbicide nitrofen (Tokyo Chemical Industry Co., LTD., Tokyo, Japan) dissolved in 1 mL of corn oil at embryonic day (E) 9.5, as previously described (Thebaud et al., 1999). Control animals did not receive any manipulation. Pregnant Sprague-Dawley rats were randomized to 2 groups: IA treatment at E13.5 (early treatment; ET) and IA treatment at E15.5 (late treatment; LT).

### Intra-amniotic sildenafil treatment

E13.5 or E15.5 timed-pregnant rats were anesthetized using inhaled isoflurane. Rats were restrained on a heated surgical platform, and a midline laparotomy was performed. Uterine horns were identified and externalized through the laparotomy incision to expose uterine saccules (fetuses). A 31G needle with a syringe was used to penetrate each uterine saccule. We injected either a 0.8 mg/mL sildenafil solution (Revatio; Pfizer, New York, NY, United States) or the same volume of phosphate-buffered saline (PBS) into the amniotic cavity. In order to determine the therapeutic range of IA sildenafil, we delivered three different doses of sildenafil to nitrofen-treated (CDH) fetuses. For the efficacy study, both CDH fetuses and control fetuses were used. The fetuses located in one uterine horn were treated with sildenafil and the fetuses located in the opposite uterine horn were treated with PBS.

The details of the dosing are shown in Table 1. Following injections, the laparotomy incision was closed, and the rats were allowed to recover.

**TABLE 1:** Dosing regimen of intra-amniotic sildenafil treatment.

Treated GD	FBW (mg)	AFV (µL)	Low dose	Therapeutic dose	High dose
E13.5 (ET)	102.2 ± 10.1	145.6 ± 31.4	10 µL (8 µg)	30 µL (24 µg)	125 µL (100 µg)
E15.5 (LT)	347.1 ± 25.3	335.0 ± 20.7	30 µL (24 µg)	100 µL (80 µg)	250 µL (200 µg)

^a^ FBW and AFV represent mean ± SD obtained from normal fetal surveillancee.

^b^ GD, gestational days; FBW, fetal body weight; AFV, amniotic fluid volume; ET, early treatment; LT, late treatment.

Close to term (E20.5), fetuses were harvested by cesarean section and assessed for various parameters, outlined below, according to our experimental design. A total of 416 fetuses underwent injection and 260 of them survived to harvest. To assess the pulmonary effect of intra-amniotic sildenafil treatment in CDH, fetuses without CDH were excluded from nitrofen-treated fetuses. Overall, a total of 133 fetuses with CDH and 58 control fetuses were analyzed (Supplementary Table S1).

### Lung morphology

We used immersion fixation for CDH lungs to reduce the artifactual effects of the injection method of lung fixation. Fetal lungs were fixed in 4% paraformaldehyde (PFA), cryoprotected in 30% sucrose overnight, embedded in Tissue-Tek O.C.T., and frozen in liquid nitrogen. Sections (5 um) were cut by cryostat and mounted. Serial sections were taken throughout both lungs and stained with hematoxylin and eosin (H&E). The stained slides were imaged using Nikon Eclipse E800 and the NIS-Elements D microscope imaging software (Nikon, Tokyo, Japan). Images were captured using ×10 and ×20 objectives. The alveolar structure was analyzed via the radial alveolar count (RAC) (Cooney and Thurlbeck, 1982) which was calculated using 3 separate locations of each lung. These 6 counts from both lungs were averaged to calculate the final RAC, and five to eight animals were used per group.

### Medial wall thickness

Histologic sections were analyzed via light microscopy. Preacinar resistance arterioles between 30 and 60 µm were included. We measured the external diameter (ED), internal diameter, and proportional medial wall thickness (MWT) using the formula: MWT (ED—internal diameter)/ED (Schmidt et al., 2012). Vascular measurements were obtained from both lungs equally using 5 to 9 different animals per group. A total of 275 vessels were analyzed.

### Western blot analysis

Snap-frozen lungs were homogenized on ice in RIPA buffer containing protease/phosphatase inhibitor cocktail (Cell Signaling Technology, Danvers, MA, United States of America). Samples were sonicated and centrifuged at 11000 rpm for 20 min at 4°C. Protein content in the supernatant was quantified using a BCA protein assay kit (Thermo Fisher Scientific, Waltham, MA, United States of America). Twenty micrograms of protein sample per lane were subjected to SDS-PAGE, and proteins from the gel were transferred to PVDF membranes by electroblotting. Immunodetection was performed with a rabbit anti-protein kinase G (PKG) −1 monoclonal antibody (#3248, Cell Signaling Technology) diluted 1:1000 and a rabbit monoclonal to vascular endothelial growth factor A (VEGFA) antibody (ab214424, abcam, Cambridge, UK) diluted 1:1000 overnight at 4°C. After the blots were washed to remove unbound antibody, secondary antibody, Goat anti-rabbit HRP conjugate (1:2000, Bio-Rad, Hercules, CA, United States of America) detection was applied for 1 h at room temperature. After being washed, bands were visualized using an enhanced chemiluminescence kit (SuperSignal West Femto Maximum Sensitivity Substrate, Thermo Fisher Scientific). Additionally, each gel was stripped and reprobed with actin or tubulin as a housekeeping protein to normalize for protein loading.

### Barium-gelatin arteriograms, Modified McGoon Index analysis, and cardiac measurements

The main pulmonary artery of E20.5 rat fetuses was canulated and injected by way of the right ventricle with a mixture of barium sulfate and gelatin at a temperature of 60°C. The lungs were then removed from the body cavity and submerged in 4% PFA. Fixed lungs were imaged using a computed tomography imaging system (Siemens, Munich, Germany). For quantitative assessment, the diameters of the right pulmonary artery (RPA) and left pulmonary artery (LPA) at the bifurcation, as well as the diameter of the aorta were measured using Radiant DICOM Viewer (Medixant, Poznan, Poland) to determine the Modified McGoon Index (MMI): (LPA + RPA)/descending aorta, a prognostic indicator for newborns with CDH (Suda et al., 2000). For the cardiac measurements, the lumenal volumes of the right ventricle (RV) and the left ventricle (LV) and the wall volumes of RV and LV plus septum (S) were measured using Inveon Research Workplace (Siemens Medical Solutions United States). 3D volumetric measurements were performed following segmentation in the three-view axial, sagittal, and coronal planes. The right ventricular wall volume to the left ventricular and septal wall volume ratio (RV/LV + S) and the right ventricular lumen to the left ventricular lumen ratio (LV/RV) were determined.

### Visualization of fetal pulmonary blood flow via *in utero* cardiac injection

Prior to fetal harvest, we performed an *in utero* intracardiac injection of fluorescein-conjugated tomato-lectin (TL) for CDH fetuses *in utero* as previously described (Shah et al., 2011). An ultrasound microscope probe was used to guide the injection apparatus. Sub-microliter injection system (Nanofil-100, World Precision Instruments, Sarasota, FL, United States of America) was used to inject TL into the heart. Each fetal heart was injected with 40 μL of TL. The injected fluorescent-conjugated lectin was allowed to circulate for 10 min to ensure adequate binding to the vascular endothelial wall of the vasculature, after which the fetus was harvested and fixed in 4% PFA for immunofluorescent analyses.

### Whole-lung immunohistochemistry with modified passive CLARITY technique (PACT)

We used the PACT protocol proposed by Yang et al. (Yang et al., 2014) with minor modifications. Following harvest, the lungs were fixed in 4% PFA at 4°C overnight. Fixed lungs were incubated at 4°C overnight in a hydrogel monomer solution A4P1 (4% acrylamide and 1% paraformaldehyde in PBS) supplemented with 0.25% photoinitiator 2,20-Azobis[2-(2-imidazolin-2-yl) propane] dihydrochloride (VA-044, Wako Chemicals United States of America, Richmond, VA, United States). A4P0-infused samples were degassed and then incubated for 2–3 h at 37°C to initiate tissue-hydrogel hybridization. After removing excess hydrogel, tissue-hydrogel matrices were embedded in a cassette and submerged in 8% sodium dodecyl sulfate (SDS) in 0.1MPBS (pH 7.5), and depending on tissue size, were incubated for 2–3 days at 37°C. For immunostaining, PACT-processed samples were washed in PBS followed by protein blocking with 10% normal donkey serum for 4 h at room temperature. Then, the samples were incubated with anti-PECAM (platelet and endothelial cell adhesion molecule) −1 antibody (Santa Cruz Biotechnology, Dallas, TX, United States) diluted 1:50 in PBST containing 10% normal donkey serum for 2 days at 4°C. Unbound antibody was removed via PBS washes, with 5 buffer changes over the course of a day, and then samples were incubated with secondary antibody (Fabfragment secondary antibody, 1:200) for 2 days at 4°C. Samples were then washed in PBS prior to incubation in imaging media (refractive index matching solution, RIMS), the details of which were described by (Yang et al., 2014). Samples were incubated in RIMS for 1–3 days at 4°C until transparent.

### Pulmonary blood flow analysis

Cleared tissue samples were mounted in RIMS with Nunc^®^ Lab-Tek^®^ Chamber Slide System (Millipore Sigma, Burlington, MA, United States). The samples were imaged by STELLARIS 5 (Leica microsystems, Buffalo Grove, IL, United States of America) under a ×10 objective. four to six images were obtained from the peripheral region from both lungs equally for each fetus using 5 different animals per group. Image reconstructions were performed using Imaris imaging software (Bitplane, Belfast, UK) with an image size of 901 μm × 901 μm x 200 μm, and blood perfusion was determined using the following formula: overlapped volume of TL to PECAM-1/total PECAM-1 volume per field (%). A total of 107 images were analyzed.

### Statistical analysis

Statistical analysis was performed using GraphPad Prism software (Prism 9, GraphPad Software, San Diego, California, United States). Values were expressed as mean ± SD. Comparisons of parameters between 2 groups were made by unpaired 2-tailed *t*-test. Comparisons between multiple groups were made using 1-way ANOVA (with repeated measures where appropriate) followed by the Holm-Šidák test for multiple comparisons. *p* < 0.05 was considered statistically significant.

The authors had full access to and take full responsibility for the integrity of the data.

## Results

### A therapeutic (mid-range) dose of sildenafil at E13.5 worked best for lung development in CDH rats

**FIGURE 1:**
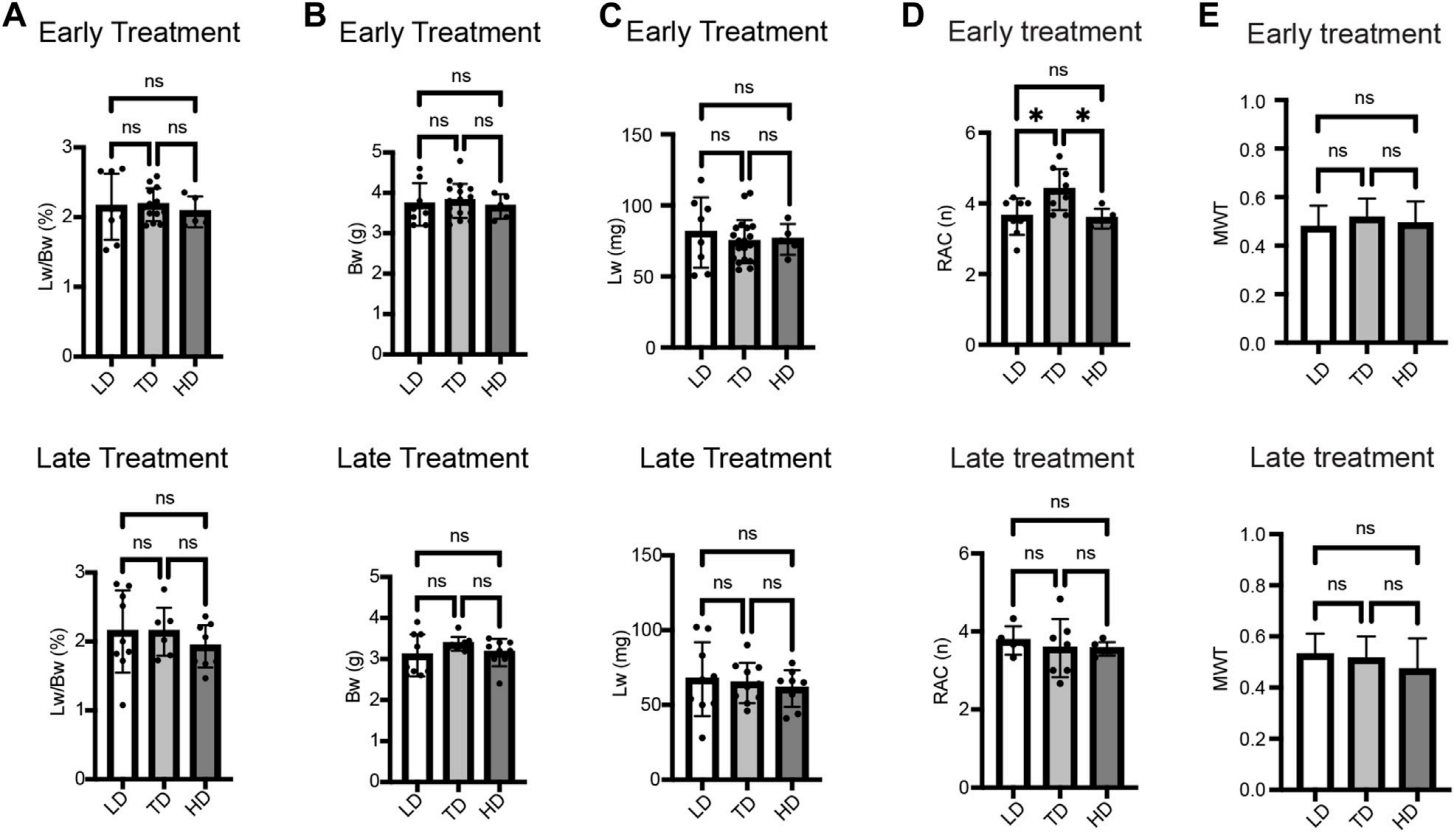
Dose-response effect of IA sildenafil treatment. **(A–C)** There was no dose-response difference in lung weight-to-body weight ratio (Lw/Bw), body weight, and lung weight in CDH fetuses after either early treatment (ET) or late treatment (LT). **(A)** Lw/Bw for 3 different Sildenafil doses (one-way ANOVA followed by Holm-Šidák test for multiple comparisons, ET: F_2,23_ = 0.1769, *p* = 0.8390; LT F_2,22_ = 0.6669, *p* = 0.5234) (n = 5–13 fetuses per group). **(B)** Body weights for the 3 different doses (one-way ANOVA followed by Holm-Šidák test for multiple comparisons, ET: F_2,27_ = 0.2539, *p* = 0.7776; LT F_2,24_ = 1.440, *p* = 0.2568) (n = 5–17 fetuses per group). **(C)** Lung weights for the 3 different doses (one-way ANOVA followed by Holm-Šidák test for multiple comparisons, ET: F_2,30_ = 0.3872, *p* = 0.6823; LT F_2,25_ = 0.2865, *p* = 0.7533) (n = 5–20 fetuses per group). **(D,E)** Dose-response effect of IA sildenafil treatment on lung histology. **(D)** Radial alveolar count (RAC) comparison between the 3 different doses (n = five to nine fetuses per group). In the ET group, the mid-range dose significantly increased RAC compared with the low or the high dose (one-way ANOVA followed by Holm-Šidák test for multiple comparisons, ET: F_2,19_ = 6.385, *p* = 0.0076; LT F_2,15_ = 0.2789, *p* = 0.7605, For ET, *p* values: *LD *versus* TD 0.0179, *TD *versus* HD 0.0183). **(E)** Mean wall thickness (MWT) of CDH fetal lung for the 3 different doses (one-way ANOVA followed by Holm-Šidák test for multiple comparisons, ET: F_2,81_ = 1.540, *p* = 0.2206; LT F_2,79_ = 2.458, *p* = 0.0921) (n = 23–36 vessels per group). LD, low dose; TD, therapeutic dose; HD, high dose.

Three different doses were used to examine the dose-response effect for both ET and LT. There was no significant dose-response relationship for lung weight-to-body weight ratio (Lw/Bw) which is an indicator of pulmonary hypoplasia, lung weight, and body weight (Figures 1A–C). However, in the ET group, CDH fetuses given the mid-range dose (therapeutic dose; TD) of IA sildenafil had a significantly higher RAC, used as a tool for estimation of lung maturation, than the low dose (LD) or the high dose (HD) of IA sildenafil (Figure 1D; Figure 4A; Supplementary Figure S1A). MWT, used as an indicator of pulmonary hypertension, did not show a significant difference between the three different doses for both ET and LT. (Figure 1E; Figure 5A; Supplementary Figure S1B). These results suggest that earlier intervention with TD is most effective in improving lung development, which is perhaps due to a minor toxicity of the HD and ineffectiveness of the LD.

### Early IA sildenafil treatment led to a decreased rate of fetal demise and increased lung weight-to-body weight ratio in CDH rats

**FIGURE 2:**
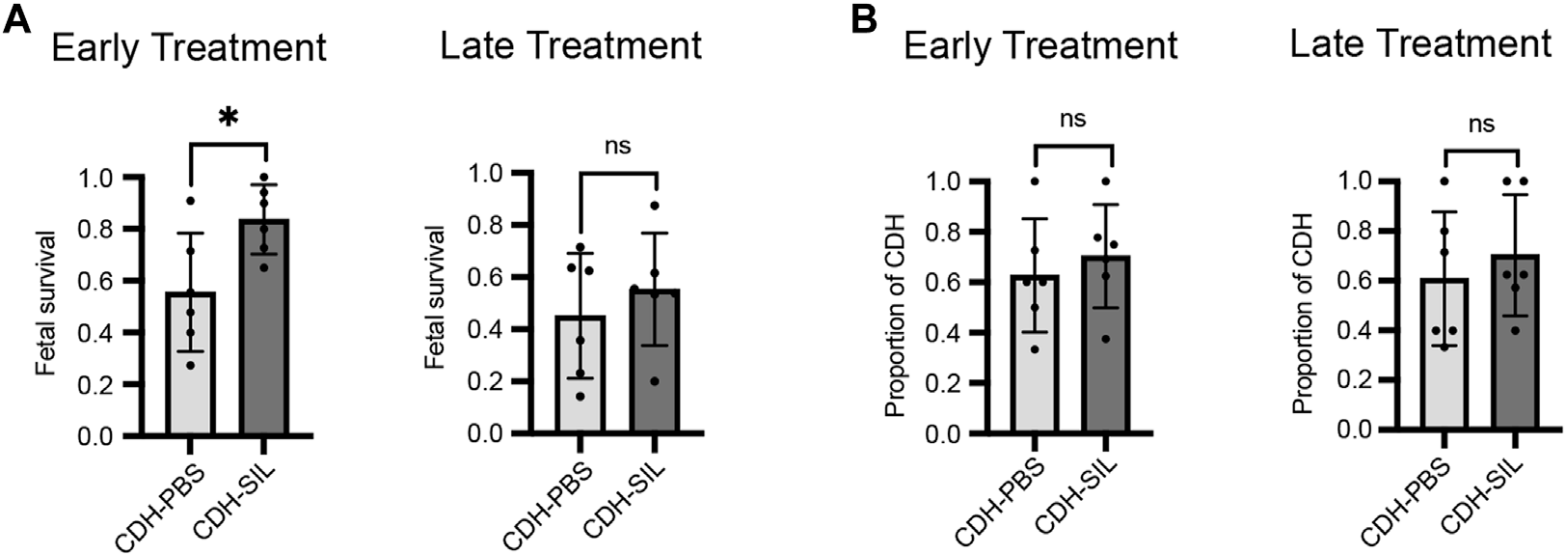
Sildenafil injection into the amniotic fluid at E13.5 improved fetal loss, with no effect on the presence of CDH. **(A)** The survival rate of IA sildenafil-treated CDH fetuses at the cesarean section near term (E20.5) was 0.83 ± 0.13 after treatment at E13.5, early treatment (ET) and 0.55 ± 0.22 after treatment at E15.5, late treatment (LT). Early IA treatment significantly improved the survival of CDH fetuses (unpaired *t*-test; *p = 0.0263). There was no difference between PBS-treated CDH fetuses and sildenafil-treated CDH fetuses on LT (unpaired *t*-test; *p* = 0.4565) (n = 6 cohorts per group). **(B)** There was no difference in the incidence of CDH between PBS-treated fetuses (CDH-PBS) and sildenafil-treated fetuses (CDH-SIL) after either ET or LT (unpaired *t*-test; *p* values: ET 0.5513, LT 0.5340) (n = 6 cohorts per group).

The mid-range dose (TD) was applied for comparison between IA sildenafil administration and IA PBS administration in order to determine the efficacy of this treatment. The survival rates of fetuses near term (E20.5) were compared in CDH fetuses treated with sildenafil or PBS (placebo) for both ET and LT. In the ET group, IA sildenafil administration led to significantly higher survival in CDH fetuses than IA PBS administration, but there was no difference between the CDH fetuses treated with sildenafil and the CDH fetuses treated with PBS in the LT group (Figure 2A). There was no significant difference in the proportion of CDH (Figure 2B) per cohort. The size of the CDH defect was also determined (Supplementary Table S2). The defect was found on the left side in 96.7% of the fetuses, two fetuses showed bilateral CDH, and only one fetus showed right CDH alone. In all groups, large “Defect C”: <50% hemi-diaphragm present, and “Defect D”: complete or near complete absence of the diaphragm, were detected in more than 70% of the fetuses. These results indicate that the improvement in fetal survival did not correlate with the defect size (Lally et al., 2013). On the other hand, IA sildenafil administration did not affect fetal survival in control fetuses. The survival after IA administration was 95% or higher in both sildenafil-treated and PBS-treated fetuses in both ET and LT in instances when the treated horn had fewer than 8 fetuses.

**FIGURE 3:**
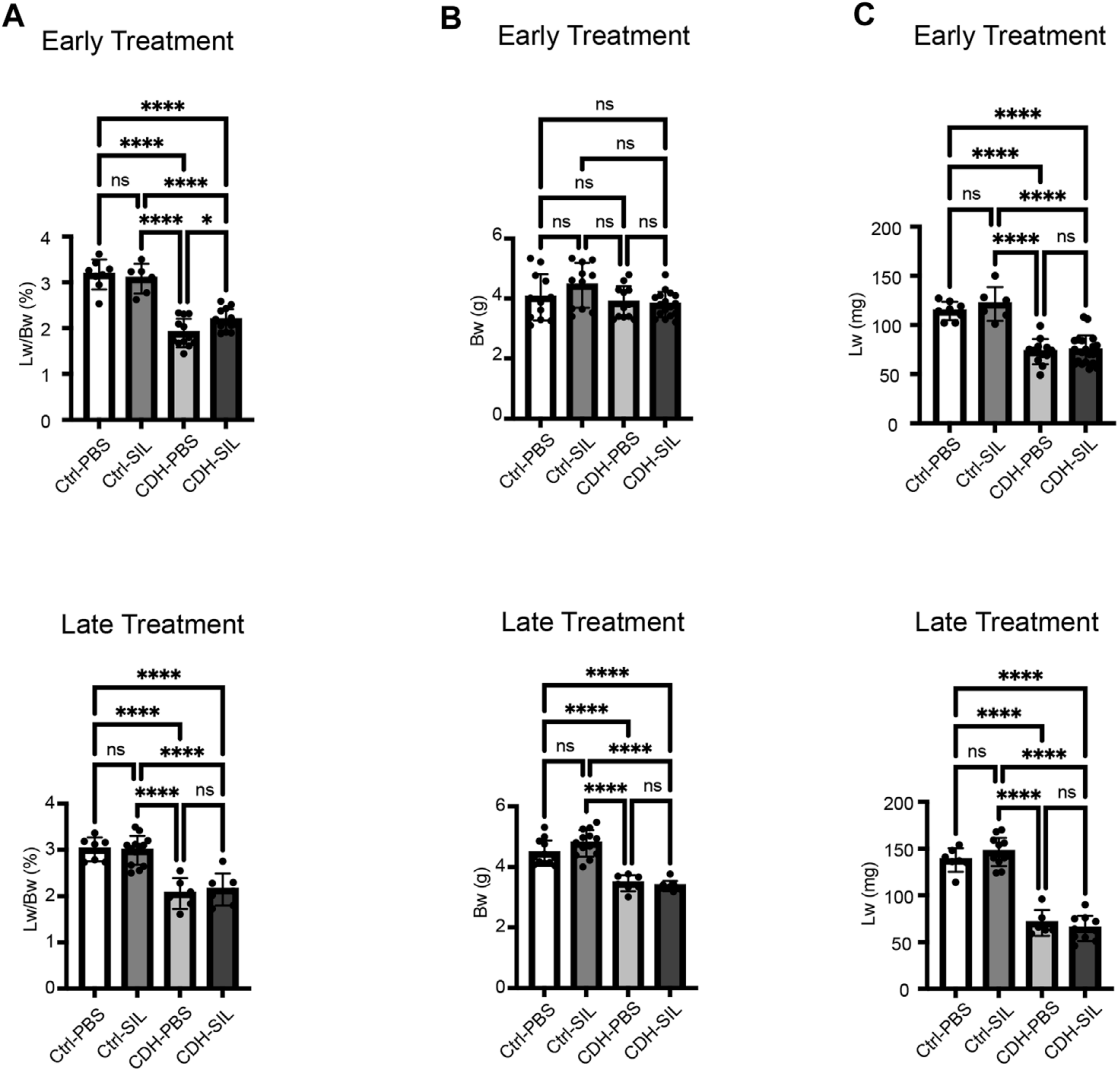
Early IA sildenafil treatment increased lung weight-to-body weight ratio in CDH fetuses with no effect on control fetuses. **(A)** Lung weight-to-body weight ratio (Lw/Bw). Lw/Bw was significantly decreased in CDH fetuses. In the ET group, Lw/Bw was significantly higher in sildenafil-treated CDH fetuses (CDH-SIL) than in PBS-treated CDH fetuses (CDH-PBS). There was no difference in Lw/Bw in the LT groups between CDH-PBS and CDH-SIL. IA sildenafil had no effect on Lw/Bw in control fetuses (one-way ANOVA followed by Holm-Šidák test for multiple comparisons, ET: F_3,35_ = 43.71, *p* < 0.0001, *p* values: * = 0.0397, ****<0.0001; LT F_3,29_ = 21.52, *p* < 0.0001, *p* values: ****<0.0001) (n = 6–16 fetuses per group). **(B)** Fetal Bw in ET and LT groups. IA sildenafil treatment did not decrease the body weight of CDH fetuses and control fetuses in both ET and LT groups. In the LT group, there were no differences between CDH fetuses and control fetuses in body weight. (one-way ANOVA followed by Holm-Šidák test for multiple comparisons, ET: F_3,48_ = 2.650, *p* = 0.0593; LT F_3,37_ = 38.21, *p* < 0.0001, *p* values: ****<0.0001) (n = 7–18 fetuses per group). **(C)** Fetal Lw in ET and LT groups. IA sildenafil treatment did not decrease the lung weight of CDH fetuses and control fetuses in both ET and LT groups. Lung weight was significantly decreased in CDH fetuses. (one-way ANOVA followed by Holm-Šidák test for multiple comparisons, ET: F_3,42_ = 32.12, *p* < 0.0001, *p* values: ****<0.0001; LT F_3,31_ = 87.47, *p* < 0.0001, *p* values: ****<0.0001) (n = 6–20 fetuses per group). Ctrl-PBS, control fetuses treated with IA PBS injection; Ctrl-SIL, control fetuses treated with IA sildenafil injection; CDH-PBS, CDH fetuses treated with IA PBS injection; CDH-SIL, CDH fetuses treated with IA sildenafil injection.

CDH fetuses had significantly reduced Lw/Bw and lung weights in both ET and LT groups (Figures 3A, C). CDH fetuses in the LT group had significantly reduced body weights, however, CDH fetuses in the ET group did not have reduced body weights (Figure 3B). Early IA sildenafil treatment increased Lw/Bw in CDH fetuses, while late IA sildenafil treatment did not significantly increase Lw/Bw in CDH fetuses. There were no differences in body weight and lung weight between sildenafil-treated and PBS-treated CDH fetuses in both treatment groups. IA sildenafil treatment had no effect on Lw/Bw, body weight, and lung weight in control fetuses. These results suggest that early IA sildenafil treatment may improve lung hypoplasia and fetal survival in CDH fetuses without negative effects in control fetuses.

### Early IA sildenafil treatment improved lung architecture in CDH rats

**FIGURE 4:**
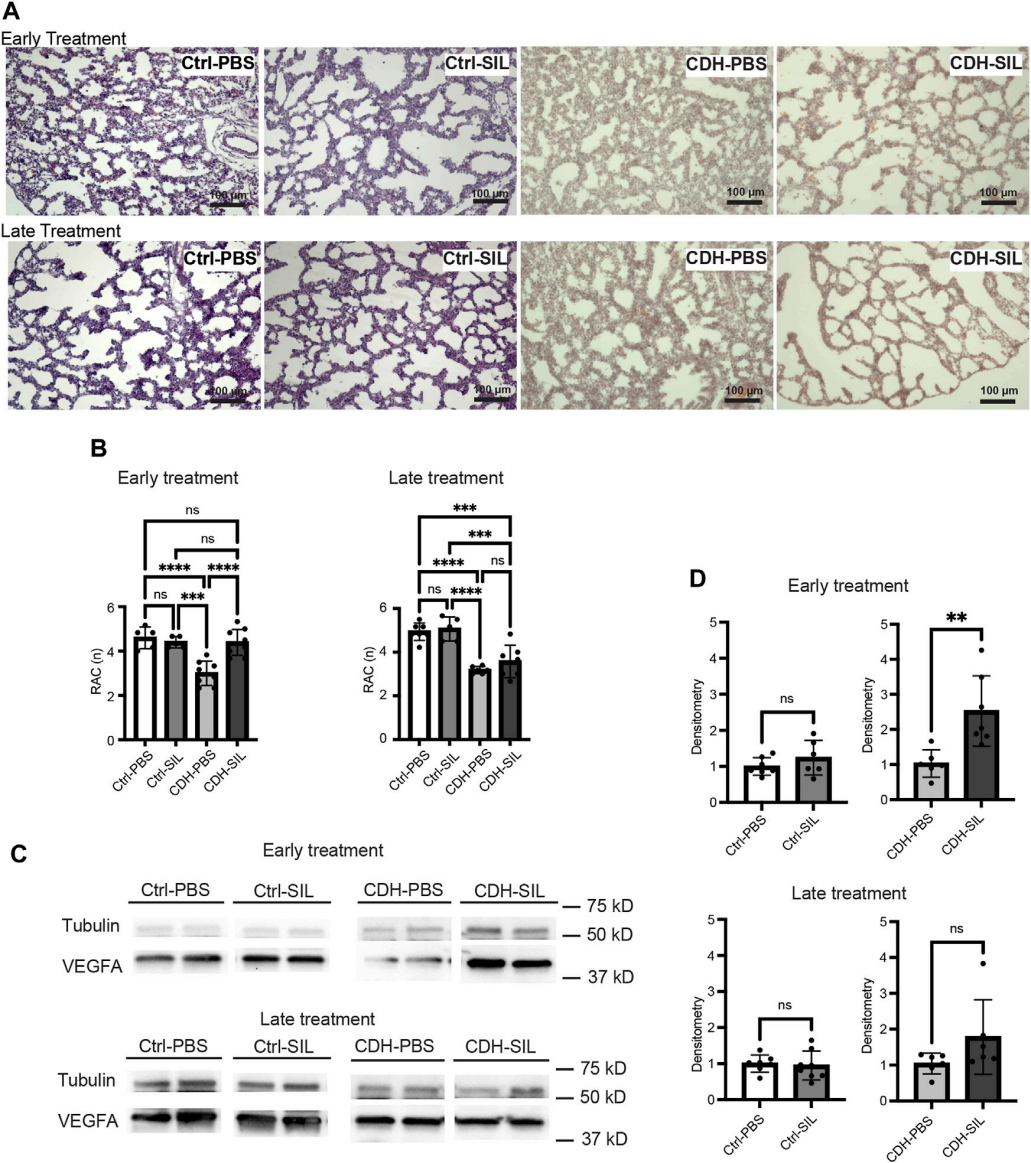
Effect of intra-amniotic sildenafil treatment on CDH lung morphometry and VEGFA expression. **(A)** Representative H&E-stained sections (100x). In both ET and LT groups, IA PBS-treated CDH fetuses (CDH-PBS) showed an apparently thinner mesenchyme compared to IA sildenafil-treated fetuses and control fetuses. **(B)** Radial alveolar count (RAC) in both treatment groups. Early IA sildenafil treatment significantly increased RAC in CDH fetuses that were similar to those of control fetuses. Late IA sildenafil treatment trended toward an increased RAC in CDH fetuses but without a statistical difference. IA sildenafil treatment had no effect on RAC in control fetuses. (one-way ANOVA followed by Holm-Šidák test for multiple comparisons, ET: F_3,23_ = 15.10, *p* < 0.0001, *p* values: *** = 0.0003, ****<0.0001; LT F_3,21_ = 20.58, *p* < 0.0001, *p* values: ***Ctrl-PBS *versus* CDH-SIL = 0.0003, ***Ctrl-SIL *versus* CDH-SIL = 0.0003, ****<0.0001) (n = five to nine fetuses). **(C)** Lung VEGFA expression on Western blot. **(D)** Densitometry of **(C)** demonstrating an increase in VEGFA in IA sildenafil-treated CDH fetuses (CDH-SIL) compared with IA PBS-treated CDH fetuses (CDH-PBS) (n = six to eight lungs per group). Early IA sildenafil treatment significantly increased VEGFA expression in CDH fetuses. Late IA sildenafil treatment trended toward an increased VEGFA expression in CDH fetuses but without a statistical difference. IA sildenafil had no effect on VEGFA expression in control fetuses. (unpaired *t*-test, ET: *p* values: Ctrl-PBS *versus* Ctrl-SIL 0.2688, CDH-PBS *versus* CDH-SIL ** 0.0058; LT: *p* values: Ctrl-PBS *versus* Ctrl-SIL 0.7723, CDH-PBS *versus* CDH-SIL 0.1246). Ctrl-PBS, control fetuses treated with IA PBS injection; Ctrl-SIL, control fetuses treated with IA sildenafil injection; CDH-PBS, CDH fetuses treated with IA PBS injection; CDH-SIL, CDH fetuses treated with IA sildenafil injection.

CDH fetuses treated with PBS showed less complexity ofairway branching and significantly lower RAC than control fetuses in both ET and LT groups (Figures 4A, B). IA sildenafil treatment significantly increased RAC in CDH fetuses in the ET group to levels similar to those of controls. In the LT groups, RAC trended higher in IA sildenafil-treated CDH fetuses compared to IA PBS-treated CDH fetuses but did not show a statistical difference. IA sildenafil treatment led to a 2.5-fold increase in VEGFA levels in CDH fetuses after ET, a crucial factor in the development of the alveolar capillary bed (Thebaud et al., 2005), and a 1.7-fold increase in CDH fetuses after LT (Figures 4C, D). IA sildenafil treatment had no effect in control fetuses on either RAC or VEGFA expression in both ET and LT groups.

### IA sildenafil treatment decreased peripheral vascular muscularization of CDH fetal lungs

**FIGURE 5:**
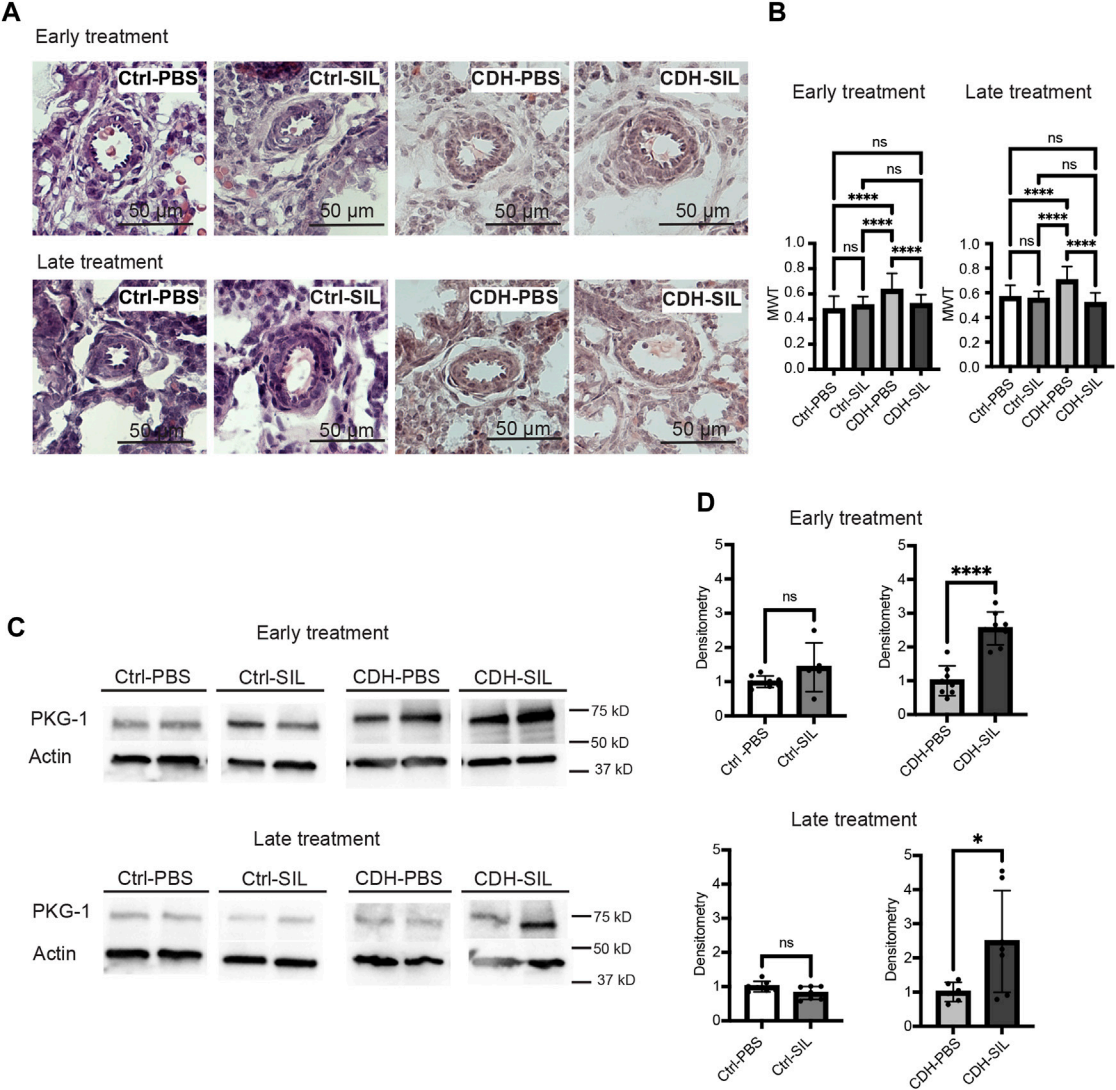
IA sildenafil treatment decreases the mean wall thickness (MWT) of CDH fetal lungs with increased PKG-1 expression in CDH fetal lungs. **(A)** Representative H&E-stained sections (200x) showing peri-acinar resistance arterioles. **(B)** IA sildenafil treatment significantly reduced MWT of CDH fetal lungs with no effect on control fetal lungs in both ET and LT groups. (one-way ANOVA followed by Holm-Šidák test for multiple comparisons, ET: F_3,122_ = 14.10, *p* < 0.0001, *p* values: ****<0.0001; LT F_3,113_ = 20.99, *p* < 0.0001, *p* values: ****<0.0001) (n = 23–39 vessels per group). **(C)** Lung PKG-1 expression on Western blot. **(D)** Densitometry of **(C)** demonstrating an increase in PKG-1 in IA sildenafil-treated CDH fetuses (CDH-SIL) compared with IA PBS-treated CDH fetuses (CDH-PBS) in both ET and LT groups. IA sildenafil had no effect on PKG-1 expression in control fetuses. (unpaired *t*-test, ET: *p* values: Ctrl-PBS *versus* Ctrl-SIL 0.1304, CDH-PBS *versus* CDH-SIL ****< 0.0001; LT: *p* values: Ctrl-PBS *versus* Ctrl-SIL 0.0676, CDH-PBS *versus* CDH-SIL *0.0359) (n = five to eight lungs per group). Ctrl-PBS, control fetuses treated with IA PBS injection; Ctrl-SIL, control fetuses treated with IA sildenafil injection; CDH-PBS, CDH fetuses treated with IA PBS injection; CDH-SIL, CDH fetuses treated with IA sildenafil injection.

The MWT of IA PBS-treated CDH fetuses was significantly higher than control fetuses in both ET and LT groups (Figures 5A, B). IA sildenafil treatment markedly reduced MWT of CDH fetal lungs that were similar to those of control fetuses in both ET and LT groups. There was no difference between IA PBS-treated control fetuses and IA sildenafil treated control fetuses.

The biological activity of sildenafil in fetal lungs was confirmed by measuring PKG -1 (Figure 5C) in fetal rat lungs. IA sildenafil treatment significantly increased PKG-1 expression in CDH fetuses 2.6-fold in the ET group and 2.5-fold in the LT group (Figure 5D). IA sildenafil had no effect on PKG-1 expression in control fetuses in both ET and LT groups. These results indicate that a single injection of IA sildenafil maintained increased PKG-1 expression in the CDH lung for a week and ameliorated the vascular hypermuscularization associated with CDH without negatively affecting control fetuses.

### IA sildenafil treatment reduced the predicted degree of pulmonary hypertension (Modified McGoon Index) in CDH rats

**FIGURE 6:**
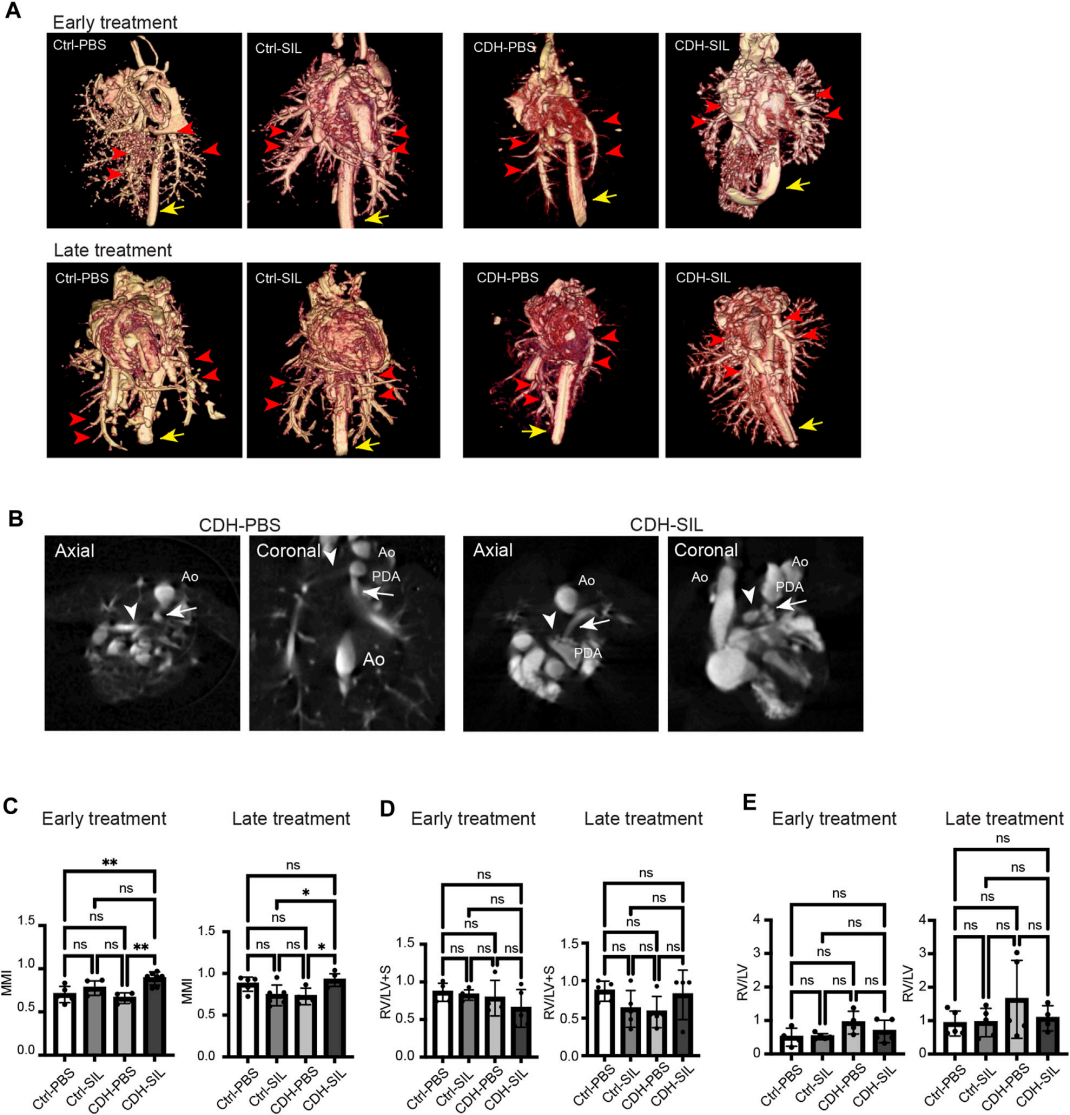
IA sildenafil treatment increased the ratio of pulmonary artery size to aortic size (MMI) in CDH rat fetuses. **(A)** Three-dimensional volume rendered images from CT angiogram of fetal lungs and hearts (red arrows, pulmonary arteries; yellow arrow, descending aorta). **(B)** Measurement views of CT for MMI. Axial and coronal planes were used (representative images from CDH-PBS and CDH-SIL in the ET group). The pulmonary artery diameters were measured at the level of bifurcation (arrowhead, right pulmonary artery; arrow, left pulmonary artery; Ao, aorta; PDA, patent ductus arteriosus). **(C)** In both ET and LT groups, IA sildenafil treatment increased MMI in CDH fetuses and had no effect on control fetuses (one-way ANOVA followed by Holm-Šidák test for multiple comparisons, ET: F_3,17_ = 8.548, *p* = 0.0011, *p* values: **Ctrl-PBS *versus* CDH-SIL 0.0057, **CDH-PBS *versus* CDH-SIL 0.0019; LT F_3,18_ = 5.453, *p* = 0.0076, *p* values: *Ctrl-SIL *versus* CDH-SIL 0.0250, *CDH-PBS *versus* CDH-SIL 0.0282) (n = four to seven fetuses per group). **(D)** The volume ratio of right ventricular wall to left ventricular wall and septum (RV/LV + S). There were no significant differences between controls and CDH fetuses. (one-way ANOVA followed by Holm-Šidák test for multiple comparisons, ET: F_3,15_ = 1.322, *p* = 0.3042; LT F_3,15_ = 1.681, *p* = 0.2135) (n = four to five fetuses per group). **(E)** The volume ratio of right ventricular lumen to left ventricular lumen (RV/LV). CDH-PBS showed higher RV/LV compared to the others but without significant differences. (one-way ANOVA followed by Holm-Šidák test for multiple comparisons, ET: F_3,14_ = 2.257, *p* = 0.1267; LT F_3,15_ = 1.196, *p* = 0.3451) (n = four to five fetuses per group). Ctrl-PBS, control fetuses treated with IA PBS injection; Ctrl-SIL, control fetuses treated with IA sildenafil injection; CDH-PBS, CDH fetuses treated with IA PBS injection; CDH-SIL, CDH fetuses treated with IA sildenafil injection.

MMI is clinically used to evaluate the risk for pulmonary hypertension in CDH fetuses, with an index of less than 1.3 indicating high risk (Suda et al., 2000). We measured the left and right pulmonary artery diameters and descending aorta diameter using a barium-gelatin angiogram and calculated an MMI in CDH rat fetuses and control fetuses (Figures 6A, B, Supplementary Video S1). IA sildenafil treatment significantly increased the MMI in both ET and LT groups (Figure 6C). IA sildenafil treatment had no significant effect in MMI on control fetuses.

We also determined the RV/LV + S as an indicator of right ventricular hypertrophy (Luong et al., 2011) and the RV/LV as an indicator of pulmonary arterial hypertension (Bax et al., 2020) (Figures 6D, E). In both ET and LT groups, there were no significant differences in RV/LV + S between controls and CDH fetuses. RV/LV trended higher in IA PBS-treated CDH fetuses compared to IA sildenafil-treated CDH fetuses and controls but did not show a statistically significant difference.

### IA sildenafil treatment improved peripheral pulmonary blood flow in CDH rats

**FIGURE 7:**
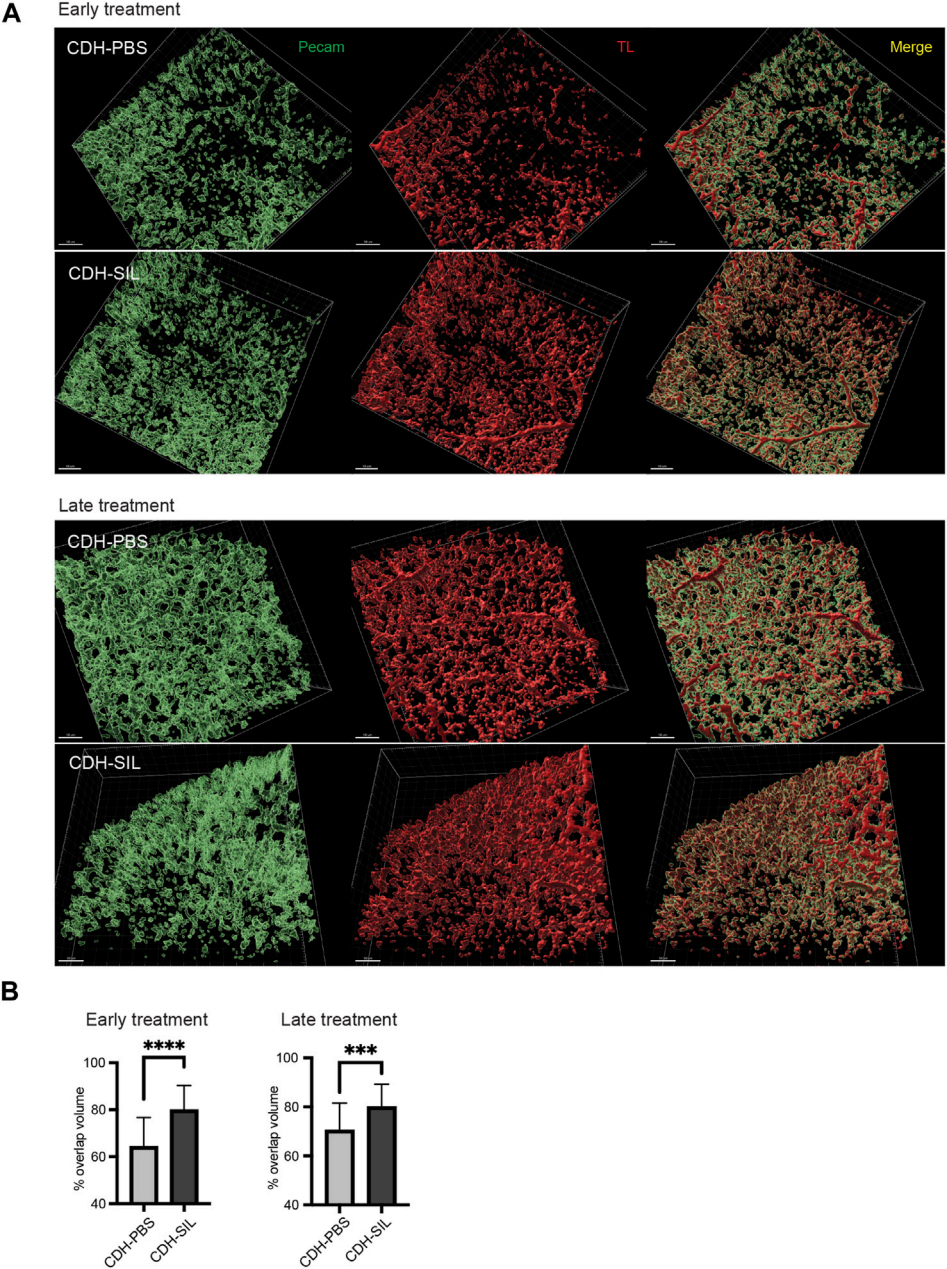
IA sildenafil treatment increased the peripheral pulmonary blood flow in CDH rats. **(A)** Reconstructed 3D images of CDH lungs. In both ET and LT groups, IA sildenafil-treated CDH lungs showed a large area of perfused vessels (TL+/Pecam + double positive) (scale bar, 100 µm). **(B)** IA sildenafil treatment significantly increased the peripheral pulmonary blood flow in CDH rats (unpaired *t*-test, ET: *p*-value: ****CDH-PBS *versus* CDH-SIL <0.0001; LT, *p*-value: ***CDH-PBS *versus* CDH-SIL 0.0009) (n = 25–29 images per group). CDH-PBS, CDH fetuses treated with IA PBS injection; CDH-SIL, CDH fetuses treated with IA sildenafil injection.

For further investigation of IA sildenafil’s effect on CDH fetuses, we visualized pulmonary blood flow in CDH fetuses. We used an *in utero* intracardiac fetal injection of a fluorescein-conjugated TL to label endothelial cells that are being perfused. The fetal heart pumps this tracer throughout the fetus to delineate which vessels are being perfused with blood, as opposed to existing blood vessels that are not receiving blood flow. By comparing TL-marked vessels (i.e., vessels receiving embryonic blood flow) with immunostaining for a general marker of PECAM-1, we identified numerous pulmonary vessels that were not receiving blood flow (non-perfused) as previously described (Shah et al., 2011). There was a clear distinction in perfused blood vessel distribution (TL+/PECAM-1+ double positive) between IA sildenafil-treated CDH fetuses and IA PBS-treated CDH fetuses (Figure 7A, Supplementary Video S2). IA sildenafil treatment significantly improved the peripheral blood perfusion of CDH fetuses in both ET and LT groups, and the improvement was more strikingly observed in the ET group (Figure 7B).

## Discussion

As prior studies have noted, interactions between the airways and blood vessels are critical during lung development (Stenmark and Abman, 2005), therefore antenatal interventions may improve both vascularization and alveolar maturation (Aman et al., 2016). Antenatal sildenafil treatment through maternal administration has been considered, with some ongoing clinical trials using maternal treatment (Russo et al., 2018a; Groom et al., 2018). However, whether there is a sufficient placental transfer of sildenafil is still uncertain, especially in early/mid-gestational ages (Russo et al., 2018b). Herein, we propose the direct administration of sildenafil to the fetus via IA injection.

Although we should consider the risk of amniocentesis regarding IA treatment, the risk of miscarriage following amniocentesis is only 0.3% in humans (Salomon et al., 2019). Thus, we think that medication administration via IA injection is a safe option for prenatal therapy. There are a few reports regarding attempted experimental therapeutic IA administration of reagents, but no treatments have reached a clinical trial (Buckley et al., 2008; Takayama et al., 2019; Kinoshita et al., 2021). A major pathway for amniotic fluid resorption is fetal swallowing/breathing (210–760 mL/day) and a second absorption pathway is the intramembranous (IM) pathway (i.e., absorption from the amniotic cavity directly across the fetal skin into the fetal vessels). Transmembranous water flow (amniotic fluid diffusing directly into maternal blood) is extremely low in comparison to IM flow (Beall et al., 2007). We think that these amniotic fluid dynamics are optimal for the intra-amniotic administration of sildenafil, maximizing fetal dosing and minimizing maternal dosing. Here, we showed the efficacy of IA sildenafil treatment, including a fetal survival advantage in the ET group, without any obvious toxicity.

In this study, the IA sildenafil dose was investigated through the use of 3 different doses. IA sildenafil treatment appeared to be well tolerated at all doses given the similarities in fetal survival and body weight across the groups. We did not find a dose-dependent improvement in lung phenotype, except that we did observe that the mid-range dose showed improved airway morphology and a trend toward higher overall body weight. We did not see any obvious adverse effects of high-dose sildenafil. Since the concentration of the sildenafil solution is fixed, the higher dose necessitated a larger injected volume, but we were unable to detect any infusion-related complications, such as an increased intra-amniotic pressure. Although further assessment of end-organ effects such as optical or neurological effects following IA sildenafil treatment is needed, our profile favors the mid-range dose in this CDH rat model.

We showed that a single IA sildenafil treatment increased PKG-1 expression for as long as 5–7 days post-administration. The biological importance of nitric oxide/cyclic guanosine monophosphate (cGMP)/PKG signaling in promoting vascular smooth muscle relaxation is well-known (Walter, 1989; Warner et al., 1994). PKG, a serine/threonine kinase, plays an important role in the regulation of vascular smooth cell contractility (Lucas et al., 2000; Hofmann et al., 2006). PKG activation results in vasorelaxation via several mechanisms, including the lowering of intracellular free Ca2+ levels and the desensitization of the contractile apparatus to Ca2+ (Xu and Liu, 2005). PKG-1 is the most prevalent PKG isoform expressed in the cardiovascular system, with a greater level in vascular smooth muscle cells and a lower level in endothelial cells (Feil et al., 2003; Stephens et al., 2010).

To assess the importance of gestational age for this treatment, we performed IA injections at E13.5 and E15.5 and assessed for differences in efficacy. Our results suggest that the earlier intervention at E13.5 promotes better lung development than the intervention at E15.5. At E13.5, an age at which the pleuroperitoneal fold (PPF, the diaphragm progenitor) has fully formed in normal rat development, nitrofen-induced CDH rats have a defect in the PPF, with the liver typically protruding through the defect (Babiuk et al., 2003). One study found that the earliest formed pulmonary vessels in mouse embryos are connected to the embryonic circulation, and they proposed that “distal angiogenesis” is responsible for lung vascular development, in which there is an expansion of preexisting vessels to vascularize the lung, as opposed to “vasculogenesis,” in which new vessels form from “blood lakes” in the mesenchyme (Parera et al., 2005). Based on this hypothesis, early IA sildenafil treatment may enhance the migration of the existing fetal pulmonary endothelial cells, which could thus contribute to the enhanced expansion of the network of blood vessels in the early stage of lung development. Thus, logically it would seem that IA sildenafil administration should be performed as soon as possible once the existence of CDH has been confirmed.

In this study, we also performed barium-gelatin angiograms in fetal rat lungs. Vascular casting has been used for visualization of 3-D vasculature, arterial density counts, and vessel volume as a means of quantitative assessment (Thebaud et al., 2005; Luong et al., 2011; Russo et al., 2016). Clinically, the size of fetal hilar pulmonary arteries is evaluated by echocardiography to calculate the MMI, which correlates with mortality in newborns with CDH. A retrospective study indicated that patients with an MMI of 1.3 or less had severe pulmonary hypoplasia frequently resulting in early mortality (Suda et al., 2000). To determine the effect of our treatment on the MMI, we measured the size of the main pulmonary arteries and the descending aorta using a CT angiogram following barium-gelatin injection. Our measured MMI in CDH rats showed improvement with sildenafil therapy, which was even better than control fetal values, similar to the other measured parameters. On the other hand, cardiac measurements did not show significant differences between controls and CDH fetuses probably because these cardiac features of pulmonary hypertension may not significantly develop before birth due to fetal circulation. However, it is well known that pulmonary vascular remodeling in CDH develops during the fetal period (Gupta and Harting, 2020) so that hilar pulmonary arteries may reflect the condition of CDH before birth. Thus, we think that MMI will be a good indicator to evaluate the efficacy of prenatal treatment for CDH and IA sildenafil injection should improve prognosis in humans as well.

Additionally, we investigated the effect of IA sildenafil treatment on pulmonary blood flow in CDH fetuses. Pulmonary hypertension is characterized by increased pressure in the pulmonary arteries and increased pulmonary arterial resistance with decreased pulmonary blood flow and leads to decreased oxygen levels in the blood (George et al., 2014). We believe that our findings here are the first report of peripheral pulmonary blood flow in a CDH animal model. Unlike other functional assessments such as *ex vivo* explant studies or echocardiography (Luong et al., 2011; Russo et al., 2016), our method produces a 3-dimensional visualization of vascular structures with real measurement of actual blood flow, not just vascularization. The method we used here was based on our previous study regarding mouse embryonic pancreatic blood flow (Shah et al., 2011). To achieve better visualization of peripheral blood flow, we injected an adequate volume of TL into the fetal heart under fetal heart monitoring by ultrasound because the quality of the results apparently is impacted by injection parameters (Robertson et al., 2015; Miyawaki et al., 2020). Here, we confirmed that the peripheral blood flow was well visualized by the intracardiac injection of TL and the results were consistent with the other results. We believe that this technique is applicable to the study of vascular development in other organs as well.

Our study design has some limitations. First, we did not show the pharmacokinetic profile of IA sildenafil administration in this model. However, our recent study showed the pharmacokinetics of IA sildenafil administration in normal rabbit fetuses (Yoshida et al., 2023). In that study, we found that IA sildenafil was rapidly absorbed into the fetuses and eliminated by the mother within 24 h after a single dose. Therefore, IA sildenafil will not cause sildenafil accumulation in the fetus and amniotic fluid, and even a short-term exposure of sildenafil will improve pulmonary hypertension in CDH fetuses. Second, the stage of lung development in rats is different from that in humans. Human CDH is mostly diagnosed around 20 weeks of gestation which is the canalicular phase of lung development, while rat CDH pathology is noticeable from E13.5 which is the glandular phase of lung development (Zoetis and Hurtt, 2003). Further investigation on later gestational time points will be preferred to determine the therapeutic effect of IA sildenafil on lung development as determined in another study of maternal sildenafil treatment (Mous et al., 2016). Third, we performed simple immersion fixation of the fetal lungs, rather than injection/infusion of fixative. We chose immersion in order to reduce the variability and potential for artifactual effects that might occur with the traditional injection/infusion method of delivering the fixative. Airway injection/infusion fixation preserves alveolar septal structures and capillary blood content, however, the fixed lung sections must be carefully assessed for rupture artifacts in small immature lungs (Hsia et al., 2010). Thus, we used RAC for lung morphology assessment because the results are independent of the degree of lung expansion. Our simple immersion fixation immediately after cesarean delivery preserved sections well enough to assess the RAC and small pulmonary arteries. Airway injection/infusion fixation has been used in previous CDH animal studies, and it may be interesting to compare these different methods in future studies of CDH lung morphology.

Finally, some animal studies showed that maternal sildenafil administration had negative effects on non-CDH fetuses (Luong et al., 2011; Mous et al., 2016; Russo et al., 2016), which may correlate with the results from the Dutch STRIDER trial as this trial was not investigating CDH fetuses. These studies suggest that maternal sildenafil treatment potentially harms non-CDH fetuses, so the concern about maternal sildenafil administration remains. However, IA sildenafil treatment did not show any negative effects on control fetuses in this study. These results insist that IA sildenafil treatment can avoid those negative effects that were found in the previous studies on maternal sildenafil administration, and it will be strong evidence to move this therapy forward to human trials.

## Conclusion

IA sildenafil treatment appears to ameliorate parameters of pulmonary hypertension in CDH fetal rats, increases peripheral lung blood flow, and improves MMI, an important prognostic indicator, without any negative effects on normal control fetuses. Early intervention with IA sildenafil may improve lung development by promoting distal angiogenesis. Our results might pave the way for clinical application of IA sildenafil treatment in human fetuses with CDH.

## Data Availability

The original contributions presented in the study are included in the article/Supplementary Material, further inquiries can be directed to the corresponding author.
