# Supplementary figures and images for: Intra-amniotic sildenafil treatment improves lung blood flow and pulmonary hypertension in congenital diaphragmatic hernia rats
Source: Front Bioeng Biotechnol. 2023 Jul 20;11:1195623. doi: 10.3389/fbioe.2023.1195623 (PMC10399963; doi:10.3389/fbioe.2023.1195623)

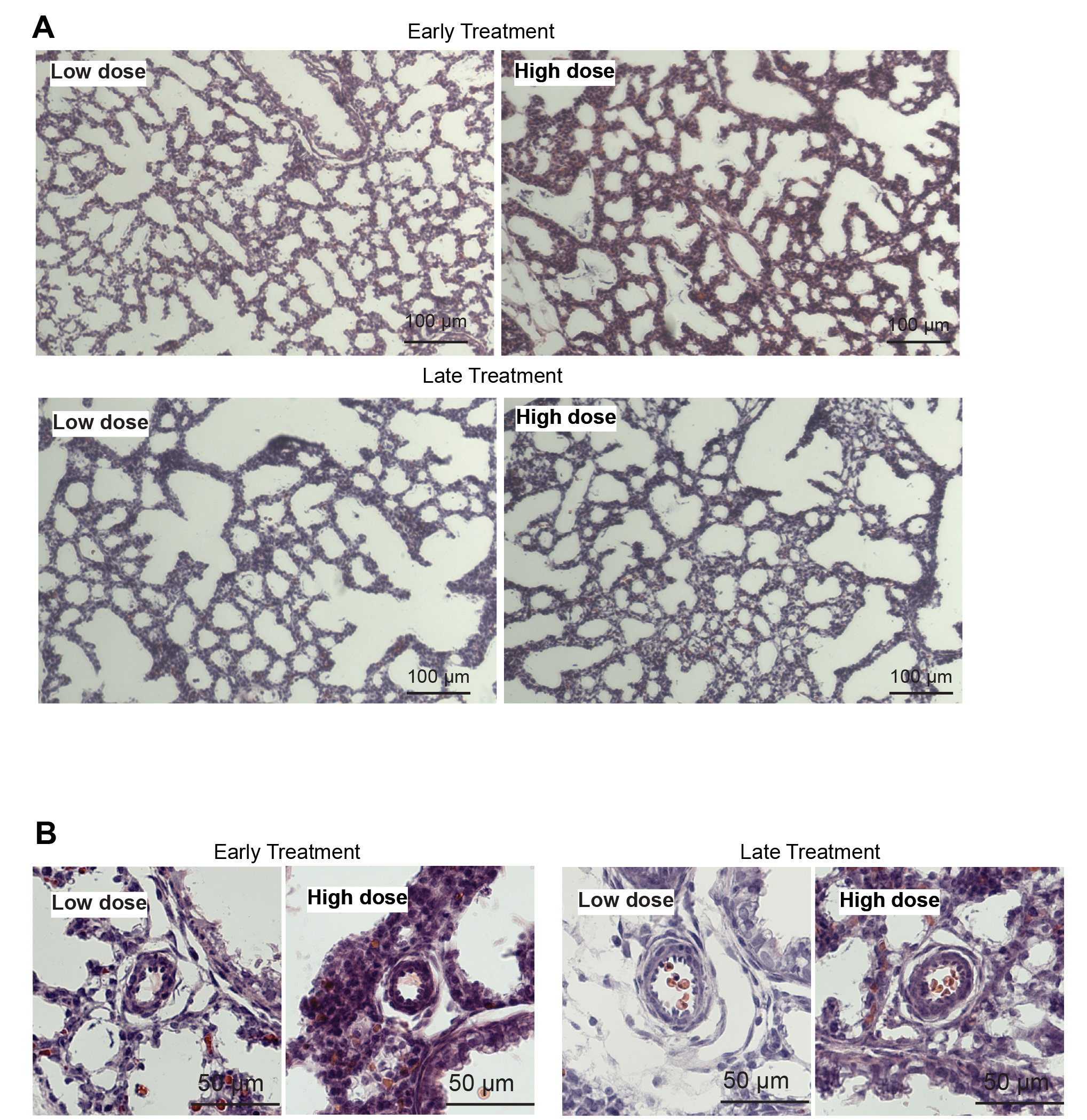

Supplement: Supplementary file 1 [file Image1.TIF]
