# Supplementary material for: Intra-amniotic sildenafil treatment improves lung blood flow and pulmonary hypertension in congenital diaphragmatic hernia rats
Source: Front Bioeng Biotechnol. 2023 Jul 20;11:1195623. doi: 10.3389/fbioe.2023.1195623 (PMC10399963; doi:10.3389/fbioe.2023.1195623)
Supplement: Supplementary file 4 [file DataSheet1.docx]

Supplementary Material

Intra-amniotic sildenafil treatment improves lung blood flow and pulmonary hypertension in congenital diaphragmatic hernia rats

**Shiho Yoshida, Alexander M. Kreger, George K. Gittes***

*** Correspondence:** George K. Gittes: gittesgk@upmc.edu

# Supplemental Table

# Supplemental Table 1. Numbers of fetuses given intra-amniotic treatment (IA), survival, and proportion of CDH in the different study groups

| Group | | IA treatment | | Survival at CS | | proportion of CDH | |
| --- | --- | --- | --- | --- | --- | --- | --- |
|  |  | ET | LT | ET | LT | ET | LT |
| Ctrl-PBS |  | 20 | 14 | 13/20 | 14/14 | - | - |
| Ctrl-SIL |  | 22 | 20 | 12/22 | 19/20 | - | - |
|  |  |  |  |  |  |  |  |
| CDH-PBS |  | 66 | 72 | 36/66 | 27/72 | 22/36 | 16/27 |
| CDH-SIL | LD | 14 | 16 | 12/14 | 11/16 | 8/12 | 9/11 |
|  | TD | 73 | 72 | 59/73 | 41/72 | 33/54 | 25/41 |
|  | HD | 6 | 21 | 6/6 | 11/21 | 5/6 | 9/11 |

# *IA treatment, intra-amniotic treatment; CS, cesarean section; CDH, congenital diaphragmatic hernia; ET, early treatment; LT, late treatment; PBS, phosphate-buffered saline; SIL, sildenafil; Ctrl, control; LD, low dose; TD, therapeutic dose; HD, high dose.

**Supplemental Table 2.** CDH phenotype of the fetuses

| IA treatment | | Defect on left side | Defect size | | | |
| --- | --- | --- | --- | --- | --- | --- |
|  |  |  | A | B | C | D |
| ET | PBS | 21/22 | 0 | 3 | 13 | 6 |
|  | SIL | 37/37 | 1 | 1 | 18 | 17 |
| LT | PBS | 13/14 | 0 | 0 | 7 | 7 |
|  | SIL | 18/19 | 1 | 4 | 9 | 5 |

*CDH, congenital diaphragmatic hernia; ET, early treatment; LT, late treatment. PBS, phosphate-buffered saline; SIL, sildenafil. Defect size, “A”: a smallest defect, entirely surrounded by muscle, “B”: a small (<50%) portion of the chest wall devoid of diaphragm tissue, “C”: a large (>50%) portion of the chest wall devoid of diaphragm tissue, “D”: complete or near complete absence of the diaphragm.

# Legends for supplemental figures and videos

Supplemental figure 1. Dose-response effect of IA sildenafil treatment on lung histology. (A) Representative H&E-stained sections (100x) from CDH fetal lungs given either the low or the high dose of IA sildenafil. (B) Representative H&E-stained sections (200x) showing peri-acinar resistance arterioles.

Supplementary Video 1. Representative reconstructed 3D image of E20.5 lung from Figure 6A in motion (from ET CDH-SIL). Video of rotating three-dimensional image allows a better appreciation of the lung vasculature.

Supplementary Video 2. Representative reconstructed 3D image of E20.5 lung from Figure 7A in motion (from ET CDH-SIL). Video of rotating three-dimensional image allows a better appreciation of the distribution of perfused vessels.
